# Root Cementum Molecular Structure and Its Role in Maintaining Oral Health—Systematic Review

**DOI:** 10.3390/ijms262211178

**Published:** 2025-11-19

**Authors:** Katarzyna Janik, Małgorzata Skucha-Nowak

**Affiliations:** Department of Dental Propedeutics, Faculty of Medical Sciences in Zabrze, Medical University of Silesia in Katowice, 15 Poniatowskiego Street, 40-055 Katowice, Poland; mskucha-nowak@sum.edu.pl

**Keywords:** cementum, biomimetics, periodontitis, root caries, guided tissue regeneration, dental implant

## Abstract

Root cementum is a specialized connective tissue with a critical role in tooth function and periodontal health. This narrative review aims to consolidate current knowledge regarding the influence of cementum on the pathophysiology of periodontitis and root caries, its remodeling during orthodontic tooth movement, its significance in regenerative strategies and implantology, along with the effect of selected stimulants. A comprehensive literature search was conducted across PubMed, Embase, and Scopus databases for articles fulfilling the inclusion criteria. The analysis revealed that cementum’s unique biological characteristics are fundamental to the success of periodontal regeneration, with biomimetic approaches showing promise for enhancing osseointegration of dental implants. Furthermore, the tissue is highly susceptible to mechanical forces, leading to adverse effects such as root resorption during orthodontic treatment. Its composition also renders it vulnerable to demineralization from root caries and damage from external stimulants. In conclusion, a profound understanding of the intricate biology of root cementum is essential for developing safer and more effective treatment modalities. The findings strongly support the development of targeted, cementum-oriented interventions and preventive strategies, thereby improving long-term success in restorative, periodontal, and orthodontic treatment.

## 1. Introduction

Cementum is one of the hard dental tissues. It is an avascular connective tissue, consisting of 65% inorganic compounds, 23% organic compounds, and 12% water. The inorganic part consists predominantly of hydroxyapatite, although other calcium compounds, such as amorphous calcium phosphate (ACP) [1], are found in this tissue in a greater quantity than in enamel and dentin [2]. The vast majority of organic compounds consists of collagen type I, representing 90% of the organic part of human cementum, while collagen type III accounts for approximately 5% [1,3]. It constitutes a scaffold for mineralization and maintains the structural integrity of mineralized cementum [4]. The subsequent most prevalent proteins are bone sialoprotein (BSP) and osteopontin (OPN), responsible for binding collagen fibrils and hydroxyapatite during mineralization and maintaining integrity afterward [5,6]. Moreover, BSP is responsible for periodontal development, including proper periodontal ligament (PDL) attachment and alveolar bone mineralization [5]. Further organic compounds include proteoglycans, such as versican, decorin, biglycan, or lumican [7,8], which inhibit the mineralization and thus their amount decreases after the course of mineralization [7]. Other proteins include cementum-derived growth factor, cementum attachment protein (CAP), or cementum protein 23 (CEMP 23) [9], which are relatively highly expressed in cementum in comparison to surrounding tissues and might be a part of markers characteristic or suggestive of cementum. However, the occurrence of none of these proteins is restricted to cementum tissue solely. Additionally, based on the study of Sarna-Boś et al., cementum presents the lowest oxygen and phosphorus content and the highest carbon and nitrogen content compared to enamel and dentin [10].

According to morphological, histological, and functional differences found along the root length, there are two main types of cementum, i.e., acellular cementum, devoid of cementocytes, located in the coronal and mid-portions of the root, and cellular cementum in the apical and inter-radicular region, with embedded cementocytes. It can be divided more thoroughly into acellular extrinsic fiber cementum (AEFC), cellular intrinsic fiber cementum (CIFC), and cellular mixed stratified cementum (CMSC). Other varieties include acellular afibrillar cementum (AAC) and intermediate cementum. AEFC covers 60–90% of the root length in single-rooted teeth and in multi-rooted teeth cervical half to one third of the length. The extrinsic collagen fibers, called Sharpey’s fibers, are integrated with principal fibers in the PDL and secreted mainly by fibroblasts and partly by cementoblasts [4]. CIFC forms an integral part of CMSC. CMSC is located in an interradicular and apical region, typically covering the apical two-thirds of the total root length [1,11,12]. It contains both extrinsic fibers, produced by fibroblasts, and intrinsic fibers, secreted only by cementoblasts. AAC is located focally just coronal to the cemento-enamel junction (CEJ). It contains neither collagen fibers nor cementocytes. Finally, the intermediate cementum remains controversial due to its origin. It is a highly calcified amorphous layer represented by the hyaline layer of Hopewell-Smith in the AEFC region [13] and by the narrow part between dentin and CMSC [14].

Moving on to the alterations that occur throughout adolescence, there are several differences between primary and secondary dentition with regard to the anatomical and histological characteristics of cementum tissue. Given that deciduous teeth undergo physiological resorption as a part of exfoliation, whereas permanent teeth’s cementum resorption occurs rarely [15], some compositional differences can be found. Giovani et al. performed a proteomic approach on non-decalcified cementum matrix to identify its protein fingerprints [16]. A total of 510 proteins were detected, of which 123 (24.1%) appeared to be unique to deciduous cementum, while 128 (25.1%) proved to be specific to permanent cementum. The remaining 259 (50.8%) proteins were found to be common to both groups. Significantly regulated proteins, such as decorin and osteocalcin (OCN) presented higher levels in permanent cementum, whereas for the deciduous group, it was, e.g., myeloperoxidase.

On the other hand, cementum is a pivotal part of periodontium, as it is fundamental for the attachment of the above-mentioned external Sharpey’s fibrils [17], which are the terminations of PDL fibrils, responsible for maintaining tooth attachment and position [5]. The regenerative potential of cementum remains unpredictable [18]. However, it is known that without newly formed, healthy cementum, no new Sharpey’s fibers can bind to the root after guided tissue regeneration (GTR) procedures. Cementoblasts seem to be able to arise from PDL cells in any area where viable dentin is exposed to them. Moreover, cementoblasts have the potential for modifying their rate of cementum deposition. The deposition of new cementum in the apical area is responsible for preserving the width of the PDL space at the apex, as well as for compensation for attrition and other occlusal relationships [19]. The cementum remodeling appears similar to the corresponding process in bone tissue; however, in contrast to bone, it seems that there is neither a direct nor predictable correlation between the functional forces applied to the tooth and the thickness of cementum [20].

The capacity for extracellular matrix synthesis and secretion enables cementoblasts to provide other functions, i.e., protection of the subjacent dentinal tubules, repair of root fractures [21,22], cemental tears [23,24,25], encasing filled canals or sealing off necrotic pulps [20].

It is the purpose of this review to focus on the role of cementum tissue in maintaining oral health, along with the destructive impact of various external and internal factors on this tissue. As cementum is associated with both pathological conditions and physiological processes related to aging, this review will examine cementum as a part of the periodontium, cementum mimicry in implantology, cementum in the development of caries, the impact of orthodontic treatment on this tissue, and the influence of selected stimulants.

## 2. Materials and Methods

### 2.1. Data Source and Search Strategy

**Figure 1:**
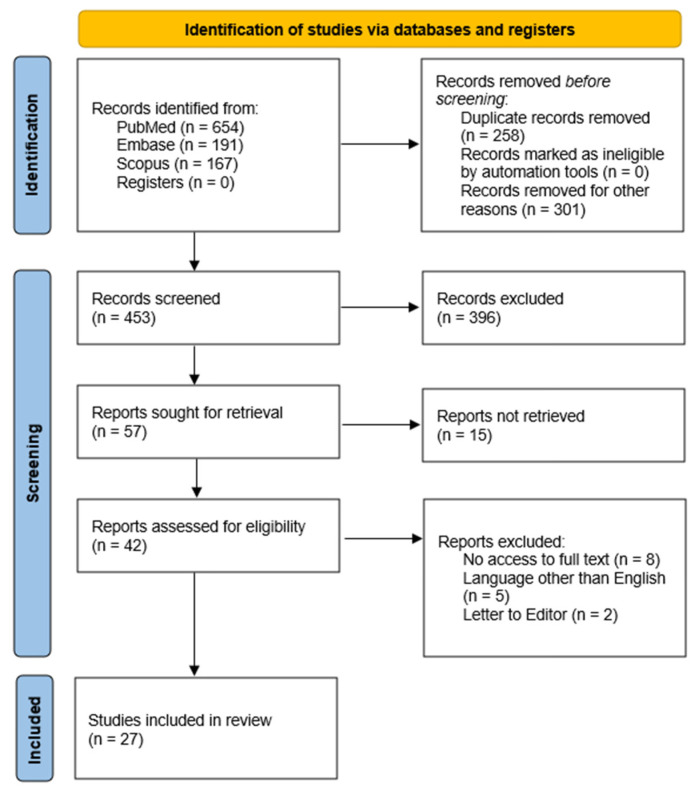
PRISMA 2020 flow diagram.

This article aimed to review the influence and relationship between root cementum tissue and root caries (RC), periodontitis, and to enhance the effectiveness of treatment using dental implants. This review adopted a more expansive approach, seeking to explore the intricate interplay between cementum tissue and the functioning of periodontium, preparation of dental implant surfaces, and RC. A comprehensive literature search was conducted in PubMed, Embase, and Scopus databases using syntaxes consisting of keywords and Boolean operators: (“cementum’’ OR “dental cementum”) AND (“periodontitis” OR “dental implant” OR “gtr” OR “orthodontic” OR “tooth movement” OR “complication” OR “root caries” OR “erosion” OR “tobacco”) adapted as appropriate for each database. A flowchart representing the research approach in accordance with the PRISMA statement [26] is displayed in Figure 1. A primary search of the databases yielded 1012 results. After the removal of duplicated studies, 453 studies were selected for the title and abstract screening, and after the screening, 411 studies were excluded as they did not fulfill the inclusion criteria. The remaining 42 studies were selected for full-text screening and out of the 42 studies, 27 articles were selected as they met the eligibility criteria and were included in the review, including 13 studies in the field of periodontitis and periodontal regeneration, 4 studies in the field of orthodontic treatment, 8 in the field of RC, and 2 in the field of selected stimulants impact on cementum.

### 2.2. Focused Questions

Focused questions included the following: 1. What role does the cementum tissue play in the function of periodontium: (a) in the physiological state; (b) in pathology; and (c) concerning regenerative dentistry? 2. How does cementum tissue react to the application of orthodontic forces? 3. What is the contribution of cementum to RC regarding: (a) its progression and (b) treatment? 4. How do selected stimulants affect the tissue of cementum, including (a) tobacco and (b) selected beverages?

### 2.3. Inclusion and Exclusion Criteria

The inclusion criteria were preclinical studies, clinical studies, case series, and reviews related to the topic of characteristics of cementum tissue in terms of periodontium functioning, periodontal regeneration, RC, complications of orthodontic treatment, and under the influence of selected stimulants. All searches were limited to articles written in English and published between 2014 and 2025.

The exclusion criteria were studies not related to cementum characteristics and their link to RC, periodontium, orthodontic treatment complications, and selected stimulants; case reports; articles in languages other than English; those with incomplete or inaccessible data; not available in full text; and published before 2014.

## 3. Results

### 3.1. Periodontitis and Periodontal Regeneration

Periodontal disease is a complex, chronic, multifactorial, inflammatory condition characterized by the degeneration and inflammation of dental cementum, PDL, gingiva, and alveolar bone [27]. The most recent worldwide classification distinguishes between four stages from I to IV and three grades from A to C [28]. The individual’s immunological dysregulation is the result of a number of different causes interacting with each other simultaneously. The major cause of such a vicious cycle is the interaction between the dysbiotic microbiome and an already dysregulated or maladaptive host inflammatory response [29,30]. Based on current literature, it is associated with several diseases, including cardiometabolic disorders, chronic kidney disease, rheumatoid arthritis, chronic obstructive pulmonary disease, chronic liver disease, and others [27,29,30,31,32,33]. The interaction between them is bidirectional, as the treatment of periodontitis has been linked to enhanced systemic health status and vice versa [33]. The standard initial treatment protocol includes complex daily hygiene maintained by the patient, combined with non-surgical periodontal therapy [34,35,36]. Both frequency and duration are effective for proper plaque removal among patients suffering from periodontal disease are individual however, general recommendations are to brush teeth twice a day with a fluoride-containing toothpaste for 2 min. Interdental cleaning devices used daily are also crucial, as interproximal surfaces are not easily accessible [36]. Non-surgical periodontal treatment involves scaling and root planing at the sites of periodontal probing depth (PPD) equal to 5 mm or deeper [35]. In more severe cases, surgical periodontal treatment is necessary. The properties of cementum tissue vary between physiological states and periodontal disease, which was researched by Sundaram et al. Mineralised collagen fibers represent the majority in healthy cementum, in contrast to the diseased tissue. The assessment of indentation profiles revealed that a healthy tissue presented a higher resistance to plastic deformation. The hardness and modulus of elasticity had significantly higher values in healthy cementum compared to diseased cementum [37].

A biomaterial-based approach is widely applied in regenerating the tissues of the periodontium. GTR is based on creating a barrier between the periodontal defect and soft tissue to facilitate the migration of slow-growing bone cells, PDL cells, and cementum into the defect by inhibiting the ingrowth of gingival epithelial cells and connective tissue cells [38,39]. It is performed by inserting a polymeric material membrane, either resorbable or non-resorbable. The vital aspects for the effectiveness of GTR procedures are space-making and wound-stabilizing capabilities of the applied membranes [40]. GTR might be extended to include the use of biomaterials, of which several are reported as successful in cementum regeneration. CaF_2_ at a concentration of 20% enhanced the expression of cementogenic genes, promoting CAP, cementum protein 1, and BSP. Moreover, it stimulated PDL stem cells’ activity several-fold compared to the control group [41]. P11–4 peptide presented a proven effect on periodontal regeneration, by stimulating the deposition of collagen I, III, and fibrillin matrix, enhancing the metabolic activity of PDL fibroblasts [42], enlarging functional PDL, while impeding epithelial growth into the defect along with promoting OCN and osteoprotegerin (OPG) expression [43]. The simultaneous application of chitin, polylactic-co-glycolic acid (PLGA), and nanobioactive glass ceramic (nBGC) in a tri-layered scaffold resulted in the stimulation of cementogenic cell differentiation, cementum matrix mineralization, and osteogenic capacity in both cementum and bone layers, as well as providing mechanical stability [44]. In the study by Kibar et al., exopolysaccharide (EPS) produced by *Weissella cibaria* EIR/P2 was found to enhance cell migration, viability of PDL cells, and thus, wound healing [45]. Enamel matrix derivative (EMD) has been found to promote cementoblast migration to the wounded area, as well as to induce OCN and runt-related transcription factor 2 (RUNX2) expression [46]. Moreover, it might recruit cementoblasts to form new root cementum and thus, facilitate the formation of a new PDL [47,48,49]. Fibroblast growth factor 2 (FGF2) has been demonstrated to exert a stimulatory effect on the formation of new cementum or cementum-like tissue [50,51,52]. Additionally, when performed on PDL fibroblasts exposed to mechanical stress, it stimulated their differentiation into cementoblasts along with their mineralization [53]. A summary of the key points from each article is presented in Table 1.

Owing to the unique, complex characteristics of cementum tissue, it is believed that it plays a crucial role in periodontal tissue regeneration. However, it remains a significant challenge for biotechnology to produce either a tissue resembling cementum or materials that promote its physiological regeneration. Several approaches were proposed, leading to promising results. The application of metastable calcium phosphates, such as ACP, provides the capability for forming cementum or cementum-like tissue [54]. Manufacturing biomimetic cementum creates entirely new possibilities for successful GTR procedures. An in vitro study revealed that biomimetic cementum significantly enhances the proliferation, cementogenic differentiation, and adhesion of PDL cells [55]. On the other hand, an in vivo part of the study demonstrated the cementogenesis-inducing capability of the tested samples. In the treatment of bone defects, newly formed cementum characteristics yield a more profound insight into the regeneration mechanism. In the study by Park et al., it was analyzed at 8 and 24 weeks post-surgical treatment. In both groups, the regenerated cementum tissue was represented by a narrow layer of AEFC in the supracrestal area and a wider layer of CMSC in the subcrestal zone. The new cementum was positioned directly adjacent to circumpulpal dentin, which contained ruffled areas, indicating a transient root resorption; however, it was not significant [56]. Recently, cell sheets of human dental follicle cells and PDL stem cells have been used in regenerating periodontal tissues [57]. They promoted cementogenic differentiation of PDL stem cells and consequently acceleration of new periodontal tissues formation along with the functional connection of PDL-like fibers to the tooth root and alveolar bone [58,59,60].

Moreover, cementum specifics remain of vital importance with regard to successful tooth loss management with dental implants. Due to the lack of cementum tissue around the implant, PDL formation and consequently the redistribution of occlusal forces is impaired. It leads to an increased risk of implant and bone overload and loss. However, manufacturing materials and thus tissues crystallographically similar to cementum poses a major challenge. There are several materials which may be used for implant coating, including β-tricalcium phosphate β-TCP or hydroxyapatite. Additionally, the alterations of titanium nanotopography remain of great importance in cementum mimicry as well. According to the literature, the implementation of implants with a cementum-mimetic surface results in the formation of dense fibrous tissue that binds the implant surface with bone matrix, restoring PDL to its primary architecture [61]. The regeneration of periodontium was achieved through the differentiation of endogenous PDL cells into cementoblasts along with the enhanced BSP expression and coordinated phosphate metabolism. Similar results might be achieved while implementing β-TCP on the surface of dental implants. Calcium phosphate promoted the formation of connective tissue resembling cementum and PDL [62]. Cementum surface characteristics might also enhance the success rate of treatment using dental implants with regard to soft tissue behavior. Generally, in a transgingival wound healing environment, gingiva-derived fibroblasts and gingiva-derived keratinocytes compete for implant surface colonization, while the first course is preferred during treatment. Bellon et al. found that applying a titanium implant surface with a roughness resembling cementum resulted in almost complete overgrowth of fibroblasts with a selective inhibition of keratinocyte colonization, in contrast to an enamel-type surface, which promoted growth of both types of cells [63]. Additionally, increasing the hydrophilicity of cementum-resembling titanium disks resulted in enhanced fibroblast adhesion, accompanied by the synthesis of a pro-regenerative environment, in contrast to such alterations in enamel-resembling titanium disks. Intensifying the hydrophilicity in cementum-type disks initiated increased expression of vinculin, aggrecan, and elastin, along with other changes in extracellular matrix content, including a decrease in collagen type I levels and an increase in collagen type V levels, leading to a decreased collagen I/collagen III ratio in the abutment microenvironment. Such a ratio is specific for scarless, regenerative healing processes [64]. The results of the above-mentioned studies are included in Table 2.

### 3.2. Orthodontic Tooth Movement

Orthodontic tooth movement (OTM) is known to induce a series of alterations in periodontal tissues, including cementum. In the study by Lira dos Santos et al., applying mesial movement to molars was observed to cause a decrease in cellular cementum volume on both the mesial side of the root, by about 10%, and the distal side of the root by about 25% [65]. Histological analysis of teeth under orthodontic forces revealed features of root resorption and counts of tartrate-resistant acid phosphatase-positive cells (TRAP+), multinucleated odontoclast/osteoclast-like cells increased three-fold in comparison to the control group, and the difference was statistically significant. Moreover, TRAP+ odontoclast/osteoclast-like cells expanded to a comparable degree on mesial and distal aspects of molar mesial roots of teeth subjected to orthodontic forces. Furthermore, alterations were also observed in the ultrastructure of cementocytes and the proteomic profile of cellular cementum. The results included a small number of empty lacunae in cellular cementum, apoptosis of cementocytes, and/or root resorption on both mesial and distal parts of the roots. OTM also induced cementocyte activation, assessed by increased nuclear sizes and proportion of euchromatin. Proteomic analysis revealed the inhibition of several extracellular matrix proteins, such as decorin, biglycan, asporin, and periostin, localized in cementum and PDL. In the cementum-dentin junction area, the most down-regulated protein was type IV collagen. On the other hand, OTM significantly enhanced eleven keratins located primarily in epithelial remnants of Hertwig’s epithelial root sheath. Similar results were observed in another study regarding TRAP+ cells on the cellular cementum, along with caspase-3- and ssDNA-positive cementocytes increase, primarily in the peripheral area, expanding by fourteen days of OTM [66]. A comprehensive analysis of the results indicates that root resorption, characterized by cementocyte apoptosis, may be induced in association with OTM.

Another pathway associated with OTM in which cementum cells participate is receptor activator of nuclear factor-κB ligand (RANKL)/receptor activator of nuclear factor-κB (RANK)/OPG. Studies by Wei et al. indicated that cementocytes may potentially function as stress receptors of the tooth in the mechanotransduction process [67,68]. It was observed that applying lower orthodontic forces resulted in downregulation of sclerostin (SOST) and OPG levels, and a lower RANKL/OPG ratio among cementocytes, with gene expression lower than in osteocytes. In contrast, in the group of higher orthodontic forces, cementocytes as well as osteocytes upregulated the SOST and RANKL/OPG ratio; however, a higher expression of SOST mRNA was observed among cementocytes.

### 3.3. Root Caries

RC is a non-cavitated or cavitated lesion below CEJ that does not involve the adjacent enamel [69]. The two phases of development of such lesions are demineralization and collagen degradation. A high consumption of fermentable carbohydrates leads to changes in oral microbiota, increasing the percentage of odontopathogens [70,71]. The initiation of RC is faster than caries in the coronal part, because dental cementum is more susceptible to demineralization in comparison to enamel [72]. This is attributable to the fact that the root surface exhibits a lower mineral and higher organic content compared to enamel, and consequently, is more vulnerable not only to demineralization but also to physical abrasion [73]. Moreover, bacterial colonization in the root area may begin earlier than in the crown, as it does not require complete demineralization due to channels of Sharpey’s fibers being possible colonization niches [71]. As RC lesions progress to more advanced stages, proteolytic enzymes have a detrimental effect on the exposed collagen, causing its fibers to lose their structural integrity [72]. However, degradation of cross-links between collagen bands might begin at the demineralization stage, owing to the activity of matrix metalloproteinases (MMPs) [74], of which MMP-13 is associated with RC [75]. Such lesions tend to be wide and shallow and spread outwards [76], increasing the unfavorable width-to-depth ratio of the cavity. Additionally, the cervical part of the root is exposed to high mechanical stress concentration. The presence of gingival crevicular fluid, contamination with blood, and a vast amount of organic material on the root surface impede moisture control [71]. These factors remain challenging in terms of restorative treatment of such lesions. Moreover, such microanatomical differences in the environment necessitate different treatment protocols from those applied in the coronal area [71,76]. According to current literature, the most effective results of RC treatment are assessed as low to moderate certainty [73,77]. It emphasizes the importance of RC treatment in the initial stages before invasive approaches become necessary.

### 3.4. Selected Stimulants

There are external factors known for their devastating impact on oral tissues, including the hard tissues of the tooth. Ankily et al. evaluated the effects of cigarettes and heated tobacco products on both enamel and cementum [78]. Each of them contains various chemicals affecting the tissues. Cigarettes contain tar, responsible for staining, and nicotine, which causes yellow discoloration of hard tissues [79,80,81]. On the other hand, heated tobacco products incorporate formaldehyde or acetaldehyde in the aerosol, which are also responsible for the color changes in the hard tissues of the tooth [82]. Furthermore, the heat generated by such devices impairs the salivary production, leading to caries development and calculus accumulation [83,84,85]. The study revealed that both cigarette smoking and the use of heated tobacco products caused significant discoloration of the hard dental tissues, as compared to the control group, with a more pronounced effect of classic cigarettes. Additionally, the cementum tissue was much more prone to the development of discoloration. The analysis of the surface showed the presence of amorphous organic structures with irregular grooves after exposure to cigarettes. On the other hand, a total loss of mosaic appearance and deep depressions were observed in the group exposed to heated tobacco products. Moreover, both types of tobacco-containing agents provoked a significant decline in calcium and phosphorus content, as well as in the Ca/P ratio.

Rajeev et al. evaluated the erosive impact of lime juice, lime soda, and carbonated beverages on enamel and cementum among both deciduous and permanent teeth [86]. The baseline microhardness of cementum in the deciduous teeth group ranged between 44.5 ± 17.9 Vickers’s hardness numbers (VHN) and in the permanent group, 50 ± 13 VHN. After immersing the teeth for one day, the microhardness of cementum in permanent teeth decreased drastically by 33.5 VHN; however, the difference was not statistically significant. At the end of ten days, a statistically significant decrease was observed in deciduous as well as permanent teeth. Maximum reduction of 41 VHN from baseline was observed in lime juice, followed by a drop of 35.8 VHN in lime soda, and finally, a decrease of 16.9 VHN in carbonated beverage. Regarding the roughness of the cementum, the maximum value equal to 2.81 was observed in lime juice, followed by 2.19 in lime soda and 1.55 in carbonated beverage. The comparison of deciduous and permanent teeth groups overall revealed that the primary dentition showed a marginally faster rate of erosion in enamel. In contrast, permanent teeth exhibited a greater loss of cementum over time.

## 4. Discussion

In this study, the results indicated the paramount importance of understanding the anatomical and physiological characteristics of root cementum in elucidating disease processes such as periodontitis, RC, as well as managing complex oral health problems, particularly those widespread among the elderly, with greater precision. This study also unveils novel prospects for therapeutic approaches that warrant further investigation.

Before drawing meaningful interpretations of the results, it is important to evaluate the limitations of the study. These include a limited number of articles, as the cementum tissue-oriented approach remains novel, and most studies focus on other hard dental tissues or elements of periodontium. This fact also contributes to the lack of meta-analyses on this topic, which has a negative impact on the reliability of the results obtained. The lack of uniformity in the research methods, sample sizes, and follow-up durations of the studies selected for analysis rendered interpretation and conclusion-drawing challenging. Restricting the search to articles written in English has also reduced the pool of articles retrieved. However, the findings of this review are predominantly based on a recent body of literature (2020–2025), which highlights the current state of research in this rapidly evolving field. The search for articles was performed independently by two authors, with broad search criteria employed to minimize the risk of omitting noteworthy articles from the review.

The increasing demand for orthodontic treatment, driven by advancements in medical technology and heightened patient awareness, has made it a common practice worldwide. As the number of treated patients grows, so does the prevalence of associated complications, including root resorption. This iatrogenic side effect, characterized by the loss of root structure, remains a significant concern and a primary limiting factor in achieving optimal treatment outcomes. As dental cementum is known to participate in both the anchoring of the PDL and the dynamic remodeling of the root surface in response to mechanical stress, a more profound and comprehensive understanding of its cellular and molecular biology is essential to mitigate adverse effects, allowing for the development of safer and more effective orthodontic treatment modalities. Further studies are essential in elucidating the factors that influence cementum and bone formation and remodeling, enabling the planning of personalized treatment plans that balance esthetic and functional goals with long-term periodontal health. This will enable the refinement of treatment algorithms while minimizing the occurrence of complications.

It has been reported that the main mediators involved in regulating bone remodeling during OTM are interleukin 1β (IL-1β), tumor necrosis factor α (TNF-α), prostaglandin E2 (PGE2), RANKL, RANK, and OPG [87,88]. The latter is a key system responsible for either osteogenesis or osteoclastogenesis initiation. Moreover, it has been found that it also regulates T-cells and T-cell-associated tissues, influencing the immune homeostasis [89]. RANKL is produced by osteoblasts, osteocytes, chondrocytes, and gingival or periodontal fibroblasts [90], and it has two isoforms: a type II membrane protein (mRANKL) and a soluble molecule (sRANKL). It is evident that both forms are bioactive; nevertheless, the membrane-bound protein appears to be the homeostatic form, whereas the production of sRANKL signals non-physiological conditions [88,91]. Its receptor, RANK, has been identified on precursor osteoclasts. The sRANKL-RANK binding initiates rapid differentiation of hematopoietic osteoclast precursors into mature osteoclasts, thereby stimulating bone resorption [90,92]. OPG is secreted by osteoblasts and osteocytes and has been identified as a decoy receptor for RANKL. By competitive binding to sRANKL, it has the ability to inhibit the differentiation of osteoclasts [93,94].

IL-1β is considered one of the most potent cytokines in the periodontal tissues during the initial phase of OTM [95]. It promotes osteoclast differentiation and exerts a multidirectional, stimulating, and overlapping effect with TNF-α. Prostaglandins, especially PGE2, have been demonstrated to directly stimulate the production of osteoclasts, thereby increasing their capacity to form ruffled borders, affecting bone resorption [95,96]. Their release occurs in response to an initial mechanical stimulus in OTM. It has been observed that applying heavy orthodontic forces stimulates RANKL expression in periodontal tissues, leading to external root resorption, in contrast to no significant changes in dental pulp tissue, preventing the development of internal root resorption [92].

A deeper insight into the intricate anatomical and physiological characteristics of cementum, particularly its microstructure and cellular composition, offers novel opportunities for enhancing the efficacy of tooth loss treatment using dental implants. A thorough understanding of this tissue’s role in maintaining tooth stability and periodontal health can be directly translated into strategies for improving osseointegration, along with peri-implant tissue regeneration. The unique ability of cementum to facilitate the attachment of PDL fibers suggests that mimicking its biological properties could lead to the development of implant surfaces that promote a more stable and biologically integrated interface. By leveraging this knowledge, there is a need for future research focusing on developing biomimetic implant coatings or materials replicating the natural connection between alveolar bone and tooth, thereby reducing the risk of implant failure and improving long-term results of treatment. Such a shift from a mechanical focus on implant integration to a biomimetic approach holds significant promise for the advancement of regenerative dentistry.

It has been demonstrated that the composition of hard tissues, including bone, cementum, and dentin, is relatively similar, as are their development mechanisms. A key stage of the maturation of these tissues is the mineralization of the extracellular matrix, regulated by a non-collagenous protein, Small-Integrin-Binding Ligand, N-linked Glycoprotein (SIBLING) family. It includes such proteins as BSP [97] or OPN. It is found that they collectively regulate the processes of hydroxyapatite mineralization and crystal growth [98]. BSP plays a significant role in osteoblast and osteoclast function, cementum initiation and/or growth, and thus, appropriate PDL structure, securing tooth attachment in the alveolar socket and maintaining proper alveolar bone composition [97,99,100,101]. During cementogenesis, BSP is associated with cementoblast differentiation and/or early mineralization of the cementum matrix, while OPN seems to inhibit mineralization during PDL formation [102]. In the study by Foster et al., it has been established that OPN performs a vital role in the regulation of dentin and bone formation and mineralization. Furthermore, its influence extends to the tissue properties of the PDL and pulp. However, OPN is not involved in the regulation of acellular cementum apposition [103].

Maeda et al. investigated the senescence of dental pulp and hard tissues of the tooth, including cementum. Alterations associated with aging of the cementum tissue include a constant increase in thickness, especially around the root apex, or a higher incidence of gingival recessions, leading to cementum exposure and an increased risk of RC and periodontal issues [104]. However, to date, no research has been conducted on the molecular mechanisms of cementum aging. Consequently, the current state of knowledge on the subject of cementum thickening regulation is limited.

In recent years, advancements in healthcare, nutrition, and public health initiatives have led to a notable increase in life expectancy across many countries. This demographic shift, characterized by a growing elderly population, presents a complex challenge. While individuals are living longer, they are also increasingly susceptible to chronic and degenerative diseases that directly affect oral health. Moreover, the population’s oral health might be affected indirectly by reduced physical dexterity, causing difficulties in maintaining proper hygiene, reduced chewing ability, leading to decreased consumption of fruits and vegetables, containing less cariogenic carbohydrates, and an increased consumption of easily chewable, however, more cariogenic carbohydrates [105,106,107]. According to the study by Atanda et al., a high level of teeth retention among the elderly is associated with better overall health, as well as quality of life [108]. However, age-related changes contribute to poor oral health, which in turn exacerbates the aforementioned symptoms [109]. Consequently, further research is needed in order to increase the effectiveness of RC and periodontitis prevention, as well as to implement minimally invasive treatment. It will serve to enhance the efficacy of therapeutic interventions for elderly patients, while concomitantly increasing the long-term effects of treatment.

## 5. Conclusions

In conclusion, this review highlights the critical role of root cementum in dental health and its involvement in various pathological processes. A deeper understanding of cementum’s biology and its response to external factors is essential for advancing clinical practices in fields of periodontology, implantology, orthodontics, and conservative dentistry. The findings underscore the need for further research to develop and implement a cementum-oriented approach, particularly in the context of an aging population.

## Figures and Tables

**Table 1:** Characteristics of the studies of biomaterials used in periodontal regeneration.

Author (Year)	Examined Factor	Results	Molecular Mechanism
Sundaram et al. (2014) [37]	Nanomechanical properties of the cervical third of the cementum in health and chronic periodontitis.	- The hardness and modulus of elasticity of the healthy cervical third cementum were significantly higher than the diseased cementum section (*p* < 0.05). - Higher resistance to plastic deformation of healthy cementum in comparison to diseased cementum (*p* < 0.05).	- The absence of mineralized cemental collagen fibers in diseased cementum. - Demineralization by organic acids of inflammatory exudates and resorption of collagen and protein polysaccharide matrix via enzyme activities within the confines of the periodontal pocket.
Liu et al. (2020) [41]	The effect of a biocompatible nanocomposite with nano-sized calcium fluoride particles on osteogenic and cementogenic induction of human PDL stem cells.	- The nano-CaF2 composite was biocompatible. - The nano-CaF2 composite supported human PDL stem cells. - The nano-CaF2 composite presented osteogenic (*p* < 0.05) and cementogenic properties (*p* < 0.05).	- Elevation of osteogenic gene expression: alkaline phosphatase (ALP)—peak at 14 days; collagen type I, runt-related transcription factor 2. (RUNX2), and OPN—peak at 21 days. - Elevation of cementogenic gene expression: CAP, CEMP1, BSP. - Bone mineral secretion was two-fold that of control (*p* < 0.05).
Koch et al. (2020) [42]	Effects of the use of self-assembling peptide P11–4 as a matrix for PDL regeneration.	P11–4 served as an efficient supporter of fibroblast activity and matrix formation in regenerative processes in PDL.	- P11–4 interacts with the human dentin surface and penetrates deeply into the dentin tubules. - Human PDL fibroblasts’ metabolic activity in the P11–4 group was 50% higher than in the control group. - P11–4 peptide matrices enhanced the deposition of collagen I, III, and fibrillin matrix around the cells.
El-Sayed et al. (2020) [43]	The effects of a self- assembling peptide P11–4 on periodontal regeneration.	Enhanced regeneration of periodontal tissues when P11–4 was used to fill periodontal defects.	- High condensation of newly formed blood capillaries directly adjacent to alveolar bone in the P11–4 group. - Highly organized fibers and oriented fibroblasts in the P11–4 group. - Superior orientation of newly formed oblique PDL fibers in the P11–4 group. - Well-constructed new bone with improved trabecular structure in the P11–4 group. - Relative epithelial down-growth significantly decreased while relative functional PDL length increased in the P11–4 group in comparison to control after 4 weeks (*p* < 0.05). - Greater levels of OPG and OCN expression and significantly higher mean OPG/RANKL ratio in the P11-4 group. - No significant differences in the proportion of proliferating cell nuclear antigen-positive cells between the P11-4 and the control group.
Sowmya et al. (2017) [44]	Regeneration of tooth supporting structures after application of tissue-specific tri-layered nanocomposite hydrogel scaffold.	The tri-layered nanocomposite hydrogel scaffold with growth factors is successful in stimulating matrix formation, mineralization in periodontal regeneration.	- Significantly enhanced collagen type I expression from day 7 to 14. - Significantly enhanced CEMP1 and BSP expression from day 7 to 21. - Significantly enhanced fibroblast surface protein expression from day 7 to 21. - Significantly enhanced RUNX2 and OCN expression; RUNX2—peak at day 7. - Significantly enhanced ALP expression. - A nearly complete mineralized matrix on the scaffold surfaces was observed on day 21.
Kibar et al. (2020) [45]	The potential of Weissella cibaria EIR/P2 EPS for periodontal regeneration.	- Weissella cibaria EIR/P2 EPS provided antimicrobial and antioxidant activity. - Weissella cibaria EIR/P2 EPS stimulated PDL fibroblasts’ proliferation.	- Significant decrease in *Streptococcus mutans* biofilm formation. - Concentration-dependent scavenging activity against 2,2-diphenyl-1-picrylhydrazyl (DPPH), superoxide radicals, and hydroxyl radicals. - Significant increase in the viability of human PDL fibroblast cells.
Mutafcilar et al. (2025) [46]	The effect of different graft materials, including Emdogain^®^, on cementoblasts’ proliferation, mineralization, and mineralized tissue-related gene expressions.	- All graft materials increased cell migration to the experimentally wounded area. - Emdogain^®^ induced OCN (*p* < 0.05) and Runx2 (*p* < 0.05) expression.	- No significant difference in cementoblasts’ proliferation at 72 h, while at 96 and 120 h all test groups showed a significant reduction in cementoblasts’ proliferation. - No significant difference in wound healing at 2 h, while at 4 h, wound healing appeared to be better in all the test groups. - On day 3 Emdogain^®^ induced OCN and Runx2 expression.
Rikimaru et al. (2025) [53]	The effects of fibroblast growth factor 2 (FGF2) and mechanical stress on PDL fibroblasts differentiation, focusing on cementoblast differentiation.	- Mechanical stress increased fibroblast growth factor receptor 1 (FGFR1) expression in PDL fibroblasts. - FGF2 promoted the differentiation of mechanically stressed PDL fibroblasts into cementoblasts and their mineralization.	- FGF2 increased the expression of CEMP1 and RUNX2. - Treatment of mechanically stressed PDL fibroblasts with FGF2 enhanced significantly the expression of FGFR1, CEMP1, CAP, and glucose transporter type 1 (GLUT1). - FGFR1 was significantly upregulated at the protein level, whereas cementoblast differentiation markers showed an upward trend after treatment of mechanically stressed PDL fibroblasts with FGF2. - At 3 weeks, no mineralization was observed; however, at 5 weeks, considerable mineralization was observed in mechanically stressed cells continuously exposed to FGF2.

PDL—periodontal ligament, ALP—alkaline phosphatase, RUNX—runt-related transcription factor, OPN—osteopontin, CAP—cementum attachment protein, CEMP—cementum protein, BSP—bone sialoprotein, RANKL—receptor activator of nuclear factor-κB ligand, OPG—osteoprotegerin, OCN—osteocalcin, EPS—exopolysaccharide, DPPH—2,2-diphenyl-1-picrylhydrazyl, FGF—fibroblast growth factor, FGFR—fibroblast growth factor receptor, GLUT—glucose transporter.

**Table 2:** Characteristics of the studies of materials used for biomimetics in periodontal regeneration.

Author (Year)	Examined Factor	Results	Molecular Mechanism
Yang et al. (2019) [59]	A combination of alternative collagen lamellae (ACL) and amorphous calcium phosphate (ACP) solution to create biomimetic cementum.	Significant promotion of the adhesion, proliferation, and cementogenic differentiation of PDL cells.	- Significant promotion of PDL cells adhesion in comparison to ACL. - Similar cell attachment efficiency with human cementum. - A potent ability of biomimetic cementum to promote cell growth at 1, 4, and 7 days. - Significant increase in CEMP1 expression. - Significant increase in BSP expression after 14 days. - There is no statistical difference in OPN expression between biomimetic cementum and human cementum at 7 and 14 days, yet significantly higher than in the ACL group. - Excellent tissue integration and no inflammation reaction surrounding the implant. - Formation of a densely packed matrix and the alternating rotation of lamellae.
Park et al. (2010) [56]	Comparison of pristine cementum and repaired cementum after surgical procedures on intrabony defect with 8 and 24-week healing period.	- Most of the repaired cementum was acellular cementum and CMSC. - Thicker layer of repaired cementum in the apical area than in the coronal area. - Amorphous acellular cementum in the supracrestal area. - The newly formed cellular cementum was partially detached from the underlying circumpulpal dentin.	- A major part of supracrestal cementum was acellular, and its shape was amorphous. - A major part of the supracrestal and notch areas was CMSC. - In some specimens, newly formed cementum extending coronally did not reach the junctional epithelium, and in these gaps, collagen fibers were attached directly to the dentin. - In the areas apical to the bone crest, the new cementum consisted of two layers: continuous acellular cementum covered by a thicker layer of CMSC. - At 8 weeks percentage of defect height was 40.94% epithelium attachment, 20.38% connective tissue attachment, and 38.69% cementum. - At 24 weeks percentage of defect height was 27.37% epithelium attachment, 5.50% connective tissue attachment, and 67.13% cementum.
Yamada et al. (2022) [61]	Smart titanium nanosurface mimicking the surface nanotopography and micromechanical properties of the tooth root cementum in periodontal regeneration.	- Formation of a complex dentoalveolar fibrous joint structure, with bone tissue, PDL, and cementum around the implant. - Regulation of matrix mineralization.	- At week 8, the TRC-mimetic titanium implants formed dense fibrous tissue connecting the implant surface and bone matrix to a level equivalent to complete recovery around the decellularized tooth, in contrast with poor fiber formation on the other titanium implants. - At week 8, TRC-mimetic titanium implants showed mineralizing signals of calcein over most of the surface in contrast to other titanium implants. - The TRC-mimetic titanium implants kept the PDL space at weeks 2 and 4 postplacement, in contrast to the loss of PDL space around other titanium implants. - At week 4, the fibrous tissue around the THC-mimetic titanium implants was oriented toward the calcein-positive implant surface and the bundle bone with aligned, active osteoblast-like cells, along with the high level of periostin, a representative matricellular molecule within PDL. - On day 10, after applying orthodontic forces, TRC-mimetic titanium implants showed a mesial displacement similar to the natural tooth and kept PDL-like spaces with the alveolar bone proper, in contrast to other titanium implants. - On day 10, after applying orthodontic forces, TRC-mimetic titanium implants activated bone remodeling on both tension and compression sides of the alveolar bone proper, in contrast to other titanium implants stimulated bone remodeling on only one side.
Safi et al. (2022) [62]	PDL restoration in osseointegrated implants coated with β-TCP using stem cells.	β-TCP-coated (titanium and zirconia) implants generated periodontal tissue and formed biohybrid implants.	- The PDL width of the titanium biohybrid implants was slightly lower than that of the natural teeth, with a statistically non-significant difference. - The PDL width of the zirconia biohybrid implants was slightly higher than that of the natural teeth, again with a statistically non-significant difference. - The mesenchymal-tissue-layered cell sheets formed natural periodontal-like tissues around β-TCP-coated implants at different intervals (45 and 90 days). - A layered-PDL cell sheet produced positive expression of periostin around the Ti and zirconia biohybrid implants at 45 and 90 days after transplantation.
Bellon et al. (2025) [63]	Testing whether titanium surface roughness disparity might be used to specifically guide the behavior of gingiva fibroblasts and keratinocytes, thereby improvingthe quality of soft tissue integration around abutments.	- Cementum-emulating titanium disk surfaces specifically impaired the growth of keratinocytes. - Cementum-emulating titanium disk surfaces sustained regular adhesion and proliferation of fibroblasts. - Increased hydrophilicity of cementum-emulating surfaces provided a more intimate adhesion between fibroblasts and titanium.	- Cementum-emulating surfaces selectively impaired the growth of keratinocytes, represented by laminin α3 levels, resulting in disks almost completely overgrown with fibroblasts, represented by fibronectin levels, in contrast to enamel-emulating surfaces stimulated the growth of both types of cells. - Titanium disks with surfaces emulating both cementum and enamel allowed a normal adhesion and morphology of keratinocytes, without any signs of material-mediated cytotoxicity. - Surface of disks with increased hydrophilicity formed more, longer, and homogeneously distributed focal adhesions all over the cell body as compared to highly localized ones at the cell borders when plated on disks without increased hydrophilicity. - Titanium disks with increased hydrophilicity stimulated the synthesis of extracellular matrix composed of reduced levels of collagen type I and of increased collagen type V, while collagen type III remained similar, resulting in a decreased collagen type I/collagen type III ratio. - Titanium discs with increased hydrophilicity stimulated the synthesis of aggrecan and elastin in the extracellular matrix, while the expression of fibronectin and tenascin was not affected by increased hydrophilicity.

PDL—periodontal ligament, ACL—alternative collagen lamellae, ACP—amorphous calcium phosphate, CMSC—cellular mixed stratified cementum, β-TCP—beta-tricalcium phosphate.

## Data Availability

No new data were created or analyzed in this study. Data sharing is not applicable to this article.

## Abbreviations

The following abbreviations are used in this manuscript:

BSP	Bone sialoprotein
OPN	Osteopontin
PDL	Periodontal ligament
CAP	Cementum attachment protein
CEMP	Cementum protein
AEFC	Acellular extrinsic fiber cementum
CIFC	Cellular intrinsic fiber cementum
CMSC	Cellular mixed stratified cementum
AAC	Acellular afibrillar cementum
CEJ	Cemento-enamel junction
OCN	Osteocalcin
GTR	Guided tissue regeneration
RC	Root caries
PPD	Periodontal probing depth
OPG	Osteoprotegerin
PLGA	Polylactic-co-glycolic acid
nBGC	Nanobioactive glass ceramic
EPS	Exopolysaccharide
ALP	Alkaline phosphatase
RUNX2	Runt-related transcription factor 2
DPPH	2,2-diphenyl-1-picrylhydrazyl
FGF	Fibroblast growth factor
FGFR	Fibroblast growth factor receptor
GLUT	Glucose transporter
ACP	Amorphous calcium phosphate
β-TCP	β-tricalcium phosphate
ACL	Alternative collagen lamellae
OTM	Orthodontic tooth movement
TRAP+	Tartrate-resistant acid phosphatase-positive
RANKL	Receptor activator of nuclear factor-κB ligand
RANK	Receptor activator of nuclear factor-κB
SOST	Sclerostin
MMPs	Matrix metalloproteinases
VHN	Vicker’s hardness numbers
IL-1β	Interleukin 1β
TNF-α	Tumor necrosis factor α
PGE2	Prostaglandin E2
SIBLING	Small-Integrin-Binding Ligand, N-linked Glycoprotein
